# A novel metabolic subtype with S100A7 high expression represents poor prognosis and immuno-suppressive tumor microenvironment in bladder cancer

**DOI:** 10.1186/s12885-023-11182-w

**Published:** 2023-08-05

**Authors:** Yun Cai, Yifei Cheng, Ziyu Wang, Lu Li, Zhengtao Qian, Wei Xia, Weiwei Yu

**Affiliations:** 1 Department of Oncology, The Affiliated Wuxi People’s Hospital of Nanjing Medical University, No.299, Qingyang Road, Wuxi, 214023 China; 2 Wuxi College of Clinical Medicine, Nanjing Medical University, Wuxi, China; 3 Department of Urology, The First Affiliated Hospital of Nanjing Medical University, Nanjing, China; 4 Department of Pathology, Nanjing Drum Tower Hospital, The Affiliated Hospital of Nanjing University Medical School, Nanjing, China; 5 State Key Laboratory of Translational Medicine and Innovative Drug Development, Jiangsu Simcere Diagnostics Co., Ltd, Nanjing, China; 6 Nanjing Simcere Medical Laboratory Science Co., Ltd, Nanjing, China; 7 Department of Clinical laboratory, Changshu Medicine Examination Institute, No.36, Qingduntang Road, Suzhou, 215500 China; 8 Department of IntensiveCareUnit, TheAffiliated Wuxi People’s Hospital of NanjingMedicalUniversity, Wuxi, China; 9 Department of Intensive Care Unit, The Affiliated Wuxi People’s Hospital of Nanjing Medical University, No.299, Qingyang Road, Wuxi, 214023 China

**Keywords:** Bladder cancer, Metabolic heterogeneity, Tumor microenvironment, S100A7

## Abstract

**Background:**

Bladder cancer (BLCA) represents a highly heterogeneous disease characterized by distinct histological, molecular, and clinical features, whose tumorigenesis and progression require aberrant metabolic reprogramming of tumor cells. However, current studies have not expounded systematically and comprehensively on the metabolic heterogeneity of BLCA.

**Methods:**

The UCSC XENA portal was searched to obtain the expression profiles and clinical annotations of BLCA patients in the TCGA cohort. A total of 1,640 metabolic-related genes were downloaded from the Kyoto Encyclopedia of Genes and Genomes (KEGG) database. Then, consensus clustering was performed to divide the BLCA patients into two metabolic subtypes according to the expression of metabolic-related genes. Kaplan-Meier analysis was used to measure the prognostic values of the metabolic subtypes. Subsequently, comparing the immune-related characteristics between the two metabolic subtypes to describe the immunological difference. Then, the Scissor algorithm was applied to link the metabolic phenotypes and single-cell transcriptome datasets to determine the biomarkers associated with metabolic subtypes and prognosis. Finally, the clinical cohort included 63 BLCA and 16 para-cancerous samples was used to validate the prognostic value and immunological correlation of the biomarker.

**Results:**

BLCA patients were classified into two heterogeneous metabolic-related subtypes (MRSs) with distinct features: MRS1, the subtype with no active metabolic characteristics but an immune infiltration microenvironment; and MRS2, the lipogenic subtype with upregulated lipid metabolism. These two subtypes had distinct prognoses, molecular subtypes distributions, and activations of therapy-related pathways. MRS1 BLCAs preferred to be immuno-suppressive and up-regulated immune checkpoints expression, suggesting the well-therapeutic response of MRS1 patients to immunotherapy. Based on the Scissor algorithm, we found that S100A7 both specifically up-regulated in the MRS1 phenotype and MRS1-tumor cells, and positively correlated with immunological characteristics. In addition, in the clinical cohort included 63 BLCA and 16 para-cancerous samples, S100A7 was obviously associated with poor prognosis and enhanced PD-L1 expression.

**Conclusions:**

The metabolic subtype with S100A7 high expression recognizes the immuno-suppressive tumor microenvironment and predicts well therapeutic response of immunotherapy in BLCA. The study provides new insights into the prognostic and therapeutic value of metabolic heterogeneity in BLCA.

**Supplementary Information:**

The online version contains supplementary material available at 10.1186/s12885-023-11182-w.

## Introduction

Bladder cancer (BLCA) is one of the most common malignancies in the urothelial system worldwide, ranking ninth in tumor incidence and thirteenth in tumor-induced mortality globally [1]. In the past few decades, platinum-based chemotherapy has been the standard-of-care first-line treatment for bladder cancer. Although approximately 60-70% patients were initially responding to platinum-based treatment, most of them will relapse and succumb to the disease due to drug resistance [2]. Moreover, despite the advances in cancer genomics, transcriptomics, proteomics, and metabolomics led to the discovery of potential biomarkers for cancers [3, 4], most of the biomarkers have failed to demonstrate superior performance characteristics compared with existing clinical tests unfortunately. Therefore, how to predict the clinical outcome more efficiently and accurately, as well as to guide the selection of adequate and sensitive treatments, is the focus of clinical research on BLCA.

The increasing number of evidence has confirmed that tumorigenesis and progression require aberrant metabolic reprogramming of tumor cells [5, 6]. Tumor cells autonomously alter their metabolic pathways to meet the increased bioenergetic and biosynthetic demand as well as mitigate oxidative stress required for tumor cell proliferation and survival [7–9]. Many studies have confirmed that compared with normal cells, tumor cells in vivo and in vitro are dependent on glycolysis for energy production and neoplastic proliferation via regulating the critical transcription factors or axis, such as HIF-α/ALYREF/PKM2 axis [10, 11]. Moreover, the aberrant metabolic reprogramming, including cholesterol metabolic pathways [12], and fatty acid metabolism [13], also resulted in tumorigenesis and progression.

Based on the advances in technology and biological science, recent works emphasize the inter- and intra-tumoral heterogeneity and flexibility of metabolism in many solid tumors [14, 15]. For example, Yu et al. revealed metabolic heterogeneity of human breast cancer by synthesizing the bulk and single-cell transcriptome profiling, and found that patients with glycolysis and pentose phosphate pathway (PPP) phenotype had a worse overall survival (OS) than those with glulaminolysis and fatty acid oxidation phenotype [16]. Moreover, the crosstalk between tumor cells and immune cells within the tumor microenvironment (TME) could affect the therapeutic response, due to the requirement of precise metabolic regulation of immune cells [17]. Preclinical studies suggest that metabolic heterogeneity within the tumor microenvironment (TME) influences local immune cell function and might contribute to treatment failures [18–21]. All findings suggested that metabolic heterogeneity plays an important part in influencing tumor progression, therapeutic vulnerabilities, and clinical outcomes. Thus, systematic and comprehensive exploration of the landscape of metabolic heterogeneity will help to understand the tumor state of patients, and provide accurate clinical prognostic information and potential therapeutic targets.

Here, we performed a systematic and comprehensive exploration of the metabolic landscape of BLCA patients, and identified promising subtype-selective metabolic vulnerabilities. In addition, combined with single-cell transcriptome profiling, we further deconstructed the TME of patients with different metabolic phenotypes to explore the underlying reasons for poor outcome, and identified potential therapeutic targets.

## Materials and methods

### Data source and preprocess

The UCSC Xena website (https://xenabrowser.net/datapages/) and the Gene Expression Omnibus (GEO) portal (https://www.ncbi.nlm.nih.gov/geo/) were used to acquire gene expression profiles of BLCA patients. After screening, GSE13507 [22] and TCGA-BLCA cohorts were obtained. The robust multi-array average (RMA) algorithm was conducted to preprocess the array profiles in the “affy” R package. After background correction, quantile normalization, and probe summarization, the gene expression profile was generated based on the platform providing gene and probe mappings. Samples with OS above one month were selected for further analysis. In addition, the immunotherapeutic bladder cancer cohort: GSE176307 [23] was also included.

### Identification of metabolic-related subpopulations for BLCA patients

In order to deconstruct the metabolic heterogeneity for BLCA patients, a total of 1,640 metabolic-related genes were downloaded from the Kyoto Encyclopedia of Genes and Genomes (KEGG) database (https://www.genome.jp/kegg/) [24]. Then, consensus clustering (the “ConsensusClusterPlus” package [25] in R, 1,000 iterations, 80% resampling) was performed to determine the optimal number of stable metabolic-related subpopulations for BLCA patients in the dataset, according to the expression of 1,640 metabolic-related genes. Finally, patients in the dataset were divided into two metabolic-related subpopulations (MRS1 and MRS2).

### Estimation of the molecular subtypes in BLCA

Several molecular subtype systems were established in previous studies, including The Cancer Genome Atlas (TCGA) [26], the Cartes d’Identité des Tumeurs (CIT)-Curie [27], MD Anderson Cancer Center (MDA) [28], University of North Carolina at Chapel Hill (UNC) [29], Lund [30], Baylor [31], and the consensus subtypes [32]. The “ConsensusMIBC” and “BLCAsubtyping” R packages were utilized to predict the molecular subtypes for each BLCA patients. In addition, twelve bladder cancer signatures that are peculiar to different molecular subtypes were acquired from the Bladder Cancer Molecular Taxonomy Group [32]. The enrichment scores of these signatures were estimated by “GSVA” R package [33].

### Assessment of the immune characteristics of the tumor microenvironment

In order to assess the immune characteristics of the tumor microenvironment (TME), immunomodulators, well-known effector genes of tumor-infiltrating immune cells (TIICs), and immune checkpoints were collected from previous studies [34, 35]. Besides, the “ESTIMATE” algorithm [36], a method inferring tumor purity and stromal or immune cell abundance from transcriptomic profiles, was applied to assess tumor purity, immune score, and stromal score.

### Prediction of therapeutic response

The therapeutic response between MRS1 and MRS2 was also assessed. According to previous research, we collected gene signatures of oncogenic pathways associated with inflamed TME, targeted therapy, and immunotherapy responses [34]. The activation scores of these pathways were calculated via “ssGSEA” function. It was notable that the mutations of several crucial genes, including TP53, RB1, ATM, ERBB2, ERCC2 and FANCC, were indicators of the response to chemotherapy in BLCA [37, 38]. Thus, we compared the mutation rates of these genes between MRS1 and MRS2 phenotypes.

### Single-cell RNA sequencing datasets acquisition and analysis

The single-cell RNA sequencing (scRNA-seq) datasets of BLCA patients were obtained from the GEO database (GSE190888 [39] and GSE186520 [40]). Quality control and pre-processing procedures was performed using “Seurat” (4.0.5, https://satijalab.org/seurat/) R toolkit [41].

To avoid the influence of abnormal cells and technical noise on downstream analysis, we removed the low-quality cells, including doublets and empty droplets. Cells were removed if the expression of mitochondrial genes was greater than 20% or with detected genes less than 200 or greater than 5,000. Finally, a total of 42,658 cells from nine BLCA patients were reserved for further analysis.

In order to minimize the technical batch effects among individuals and experiments, we used the “RunHarmony” function in R package “harmony” [42] to perform integration. The top 4,000 variable genes were used for principal component analysis (PCA) to reduce dimensionality. The dimensionality of the scaled integrated data matrix was further reduced to two-dimensional space based on the first 30 principal components (PCs) and visualized by t-Distributed Stochastic Neighbor Embedding (tSNE). The cell clusters were identified based on a shared nearest neighbor (SNN) modularity optimization-based clustering algorithm with a resolution of 3. According to the expression levels of some well-known markers, the 42,658 cells were annotated as four cell types, including epithelial cells, fibroblasts, T cells and macrophages.

### Linking cells with metabolic-related subpopulations

In order to link cells with metabolic-related subpopulations, we applied “Scissor” algorithm [43] in “Scissor” R package to identify cell subpopulations from single-cell data that are associated with a given phenotype from TCGA profiling. Firstly, we integrated TCGA-BLCA expression data and single-cell data by quantifying the similarity between each single cell and each bulk sample. Then, a regression model was optimized on the correlation matrix with the sample phenotype to identify relevant metabolic-related subpopulations.

### Evaluation of proliferation for single-cell

In order to estimate the proliferation of each single cell, we used the “CellCycleScoring” function to predict the cell cycle state according to a series of cell cycle-related signature. Furthermore, we computed the proliferation score for each single cell by using a signature consisting ten genes that were highly expressed in cycling cells (ASPM, CENPE. CENPF, DLGAP5, MKI67, NUSAP1, PCLAF, STMN1, TOP2A, TUBB) [44]. For each of these signature genes, we selected the 100 genes with the smallest difference in average expression levels as a background gene set. The average expression level of the background genes was subtracted from the respective signature gene, and the average of the yielded values of all signature genes was kept as the proliferation score.

### Cell-cell communication analysis

Cell-cell communications mediated by ligand-receptor complexes were critical to diverse biological processes, such as inflammation and tumorigenesis. To investigate the molecular interaction networks between different cell types, we used “CellPhoneDB” [45], a software to infer cell-cell communication from the combined expression of multi-subunit ligand-receptor complexes, to analyze the interactions between tumor cells and microenvironment cell subpopulations. The ligand-receptor pairs with a *P* value < 0.05 were remained for the assessment of relationship among different cell clusters.

### Identification of differentially expressed genes (DGEs)

For single-cell datasets, “FindAllMarkers” function from “Seurat” package was used to identify the specific genes of each group. For bulk datasets, the R package “limma” [46] was applied to recognize DEGs. Genes with the adjusted *P*-value < 0.05 and | fold change (FC) | ≥ 1.5 were determined as DEGs.

### Enrichment analysis of gene functions and pathways

The enrichment analysis was performed through the following steps: according to the pathway gene set of the Molecular Signatures Database in the R package “ClusterProfiler” [47], the activation degree of each pathway was calculated using GSEA [48]; then the differential pathway was identified by the “limma” package [46].

### Clinical samples

The BLCA tissue microarray (TMA, HBlaU079Su01) was obtained from Outdo Biotech (Shanghai, China). The HBlaU079Su01 microarray contained 63 BLCA and 16 adjacent samples. Ethical approval for the study of tissue microarray slides was granted by the Clinical Research Ethics Committee, Outdo Biotech.

### Immunohistochemistry and semi-quantitative scoring

Immunohistochemistry (IHC) staining was directly conducted on the HBlaU079Su01 TMA with standard procedures. The primary antibodies used were as follows: anti-S100A7 (1:100 dilution, Cat. 13061-1-AP, ProteinTech, Wuhan, China) and anti-PD-L1 (Ready-to-use, Cat. GT2280, GeneTech, Shanghai, China). Antibody staining was visualized with DAB and hematoxylin counterstain, and stained sections were scanned using Aperio Digital Pathology Slide Scanners. The stained TMA was independently assessed by two pathologists. The percentage of positively stained tumor cells was scored as 0–4: 0 (< 1%), 1 (1–5%), 2 (6–25%), 3 (26–50%) and 4 (> 50%). The staining intensity was scored as 0–3: 0 (negative), 1 (weak), 2 (moderate), and 3 (strong). The immunoreactivity score (IRS) equals the percentage of positive cells multiplied by the staining intensity.

### Cell culture and proliferation test

BLCA cell line RT4 (Cat. KG089) was obtained from KeyGEN (Nanjing, China). RT4 cells were cultured in McCoy’s 5A media added with 10% fetal bovine serum (FBS) at 37°C with 5% CO_2_. All assays were conducted with mycoplasma-free. For S100A7 inhibition, RT4 cells were transfected with siRNA obtained from Thermo Fisher (Cat. s12421) for S100A7 using Lipofectamine 3000 (Cat. L3000015, Invitrogen, CA). The transfection efficiency was validated by quantitative real‑time PCR (qRT-PCR). The primers for *S100A7* and *GAPDH* mRNA reverse transcription were synthesized in KeyGEN (Nanjing, China). The detailed information of primers used for gene amplification was shown as follows. S100A7 [49]: (forward) 5’-AACTTCCTTAGTGCCTGTG-3’, (reverse) 5’-TGGTAGTCTGTGGCTATGTC-3’; GAPDH [50]: (forward) 5’-AGATCATCAGCAATGCCTCCT-3’, (reverse) 5’-TGAGTCCTTCCACGATACCAA-3’. The functions of S100A7-knockdown RT4 cells were checked. For cell proliferation detection, CCK-8 and EdU assays were applied. CCK-8 (Cat. KGA317s) assay kit was obtained from KeyGEN (Nanjing, China) and EdU (Cat. C10310-1) assay kit was RIBOBIO (Guangzhou, China). The detailed protocol was according to the manufacturer’s protocol.

### Statistical analysis

All statistical analyses were handled using R software (version 4.0.4). The differences in continuous variables between two groups were assessed using the Wilcoxon rank-sum test, while Fisher exact test was used to measure the differences among categorical variables. Prognostic values were evaluated using the log-rank test. For all analyses, a two-paired *P*-value < 0.05 was deemed to be statistically significant, and labeled with * *P*-value < 0.05, ** *P*-value < 0.01, *** *P*-value < 0.001, and **** *P*-value < 0.0001.

## Results

### Metabolic-related stratification of BLCA patients

**Fig. 1:**
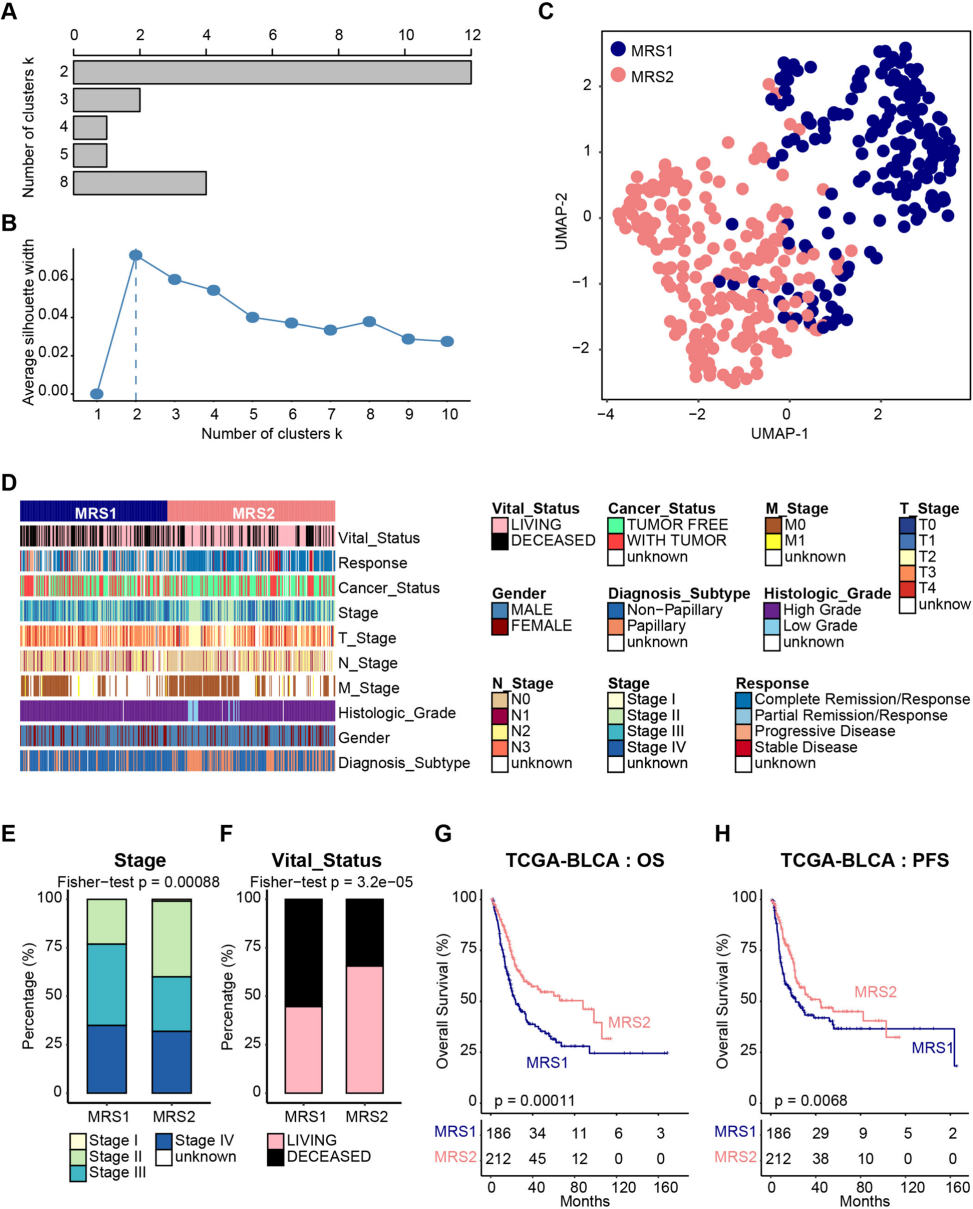
Metabolic-gene-based stratification of TCGA-BLCA patients. **A** NbClust analysis of BLCA metabolic-gene-based subtypes. **B** Silhouette analysis of clustering results. **C** UMAP visualization of metabolic subtypes in the TCGA cohort for the expression of metabolic genes. **D** Correlations between metabolic subtypes and clinicopathological features in BLCA. **E**, **F** Barplot showing the percentage of pathological stages (**E**) and vital status (**F**) in the MRS1 and MRS2 groups. **G**, **H** Kaplan-Meier analysis in term of OS (**G**) and PFS (**H**) in TCGA cohort

To deconstruct the metabolic heterogeneity of BLCA patients, we extracted 1,640 human genes assigned to 85 metabolic pathways from the KEGG database [24] (https://www.genome.jp/kegg/). Unsupervised clustering based on the expression profile of these metabolic genes showed that BLCA had distinctive metabolic gene transcriptomic levels from normal samples in the TCGA-BLCA cohort, whereas normal samples seemed to share relatively higher similarity (Supplementary Fig. 1A). The divergence in metabolic gene expression levels, measured by euclidean and correlation distance, was significantly larger both between tumor and normal and within tumor samples than within normal samples (Supplementary Fig. 1B). To reveal the metabolic heterogeneity of BLCA patients, unsupervised clustering based on the expression profile of these metabolic genes was performed. According to the consensus clustering matrixes, the number of tests supporting the cluster number from the NbClust testing, and the silhouette analysis, we identified that the optimal cluster number was two (Fig. 1A, B, Supplementary Fig. 2). Then, all 398 BLCA tumors were classified into two subtypes (MRS1 and MRS2) by using unsupervised k-means clustering based on the transcription levels of metabolic genes (Fig. 1C).

Subsequently, we evaluated the relationship between metabolic subtypes and the clinicopathological features of BLCA samples. Patients harboring MRS1 tend to develop high histological grade, higher pathological stages (Stage III and Stage IV), and nonpapillary subtype (Fig. 1D, E, Supplementary Fig. 3), and expectedly skew towards worse clinical outcomes (Fig. 1F). Moreover, survival analysis suggested that MRS1 phenotype was associated with the poor OS (Fig. 1G) and progression-free survival (PFS, Fig. 1H) of BLCA patients in the TCGA cohort. The results were validated in the GSE13507 database. A total of 165 patients were divided into two metabolic subgroups (MRS1 and MRS2, Fig. 4A); patients with the MRS1 phenotype showed a worse OS (Fig. 4B). In summary, our results indicated the metabolic heterogeneity in the BLCA tumor samples, and the prognostic values of metabolic phenotypes.

### MRS1 phenotype was associated with the immuno-suppressive microenvironment

**Fig. 2:**
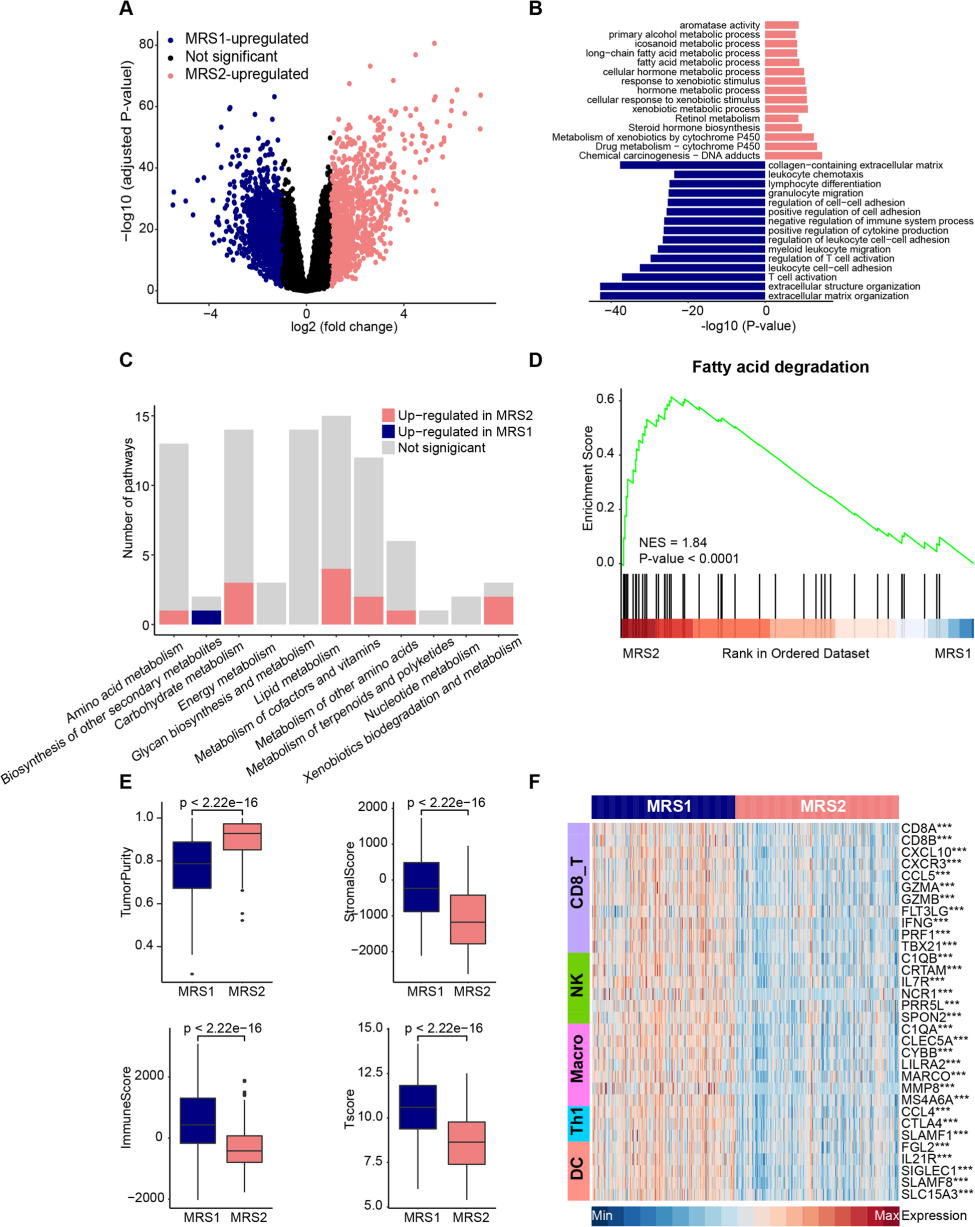
Metabolic phenotypes show distinct metabolic and immune features in the TCGA cohort. **A** Volcano plot showing the differentially expressed genes of the MRS1 and MRS2 groups. Each dot represents one gene, colored by the groups. MRS1-specific genes are represented by navy, MRS2-specific genes are represented by lightcoral. **B** Functional enrichment analysis of genes specifically expressed in MRS1 or MRS2 group. Each bar represents one pathway, colored by the groups. MRS1-upregulated pathways are represented by navy, MRS2- upregulated pathways are represented by lightcoral. **C** The number of metabolic pathways that was significantly upregulated (*p*-value < 0.05) in the MRS1 or MRS2 group in the TCGA cohort. **D** A representative gene set enrichment analysis plot showing significant upregulated fatty acid degradation in theMRS2 group versus the MRS1 group in the TCGA cohort. (**E**) Comparison of ESTIMATE results between the MRS1 and MRS2 group. **F** Expression levels of the gene markers of the common TIICs in the MRS1 and MRS2 groups. *p < 0.05, **p < 0.01, ***p < 0.0001

Next, we explored the biological characteristics of the two groups, especially the activation of metabolic-related pathways. Differential expression analysis was performed to identify the up-regulated genes in the MRS1 and MRS2 phenotypes, respectively (Fig. 2A, Supplementary Table 1). Then, functional enrichment analysis of up-regulated genes revealed the distinct functional pattern of MRS1 and MRS2 phenotypes, respectively. As shown in Fig. 2B, lipid metabolism pathways such as fatty acid metabolic processes were significantly activated in patients with MRS2 phenotype. Moreover, we also performed GSEA of metabolic pathways in MRS1 and MRS2, the results demonstrated that a total of 16 metabolic pathways, encompassing the majority of metabolic processes, were significantly up-regulated in the MRS2 phenotypes compared with the MRS1 phenotype (Fig. 2C, Supplementary Table 2), especially the pathways belong to lipid metabolism, such as fatty acid degradation (Fig. 2D). Furthermore, the up-regulated pathways in the MRS1 phenotype were enriched in several immune-related signaling pathways, including the leukocyte chemotaxis, lymphocyte differentiation, and T cell activation (Fig. 2D), indicating that patients with MRS1 phenotype tended to remodel an immune infiltrating microenvironment.

Therefore, we subsequently explored the immunological characteristics of the metabolic phenotypes. The ESTIMATE analysis showed that compared with the MRS2 phenotype, the MRS1 group exhibited higher levels of Immune Score and Stromal Score, along with lower Tumor Purity (Fig. 2E), indicating that tumors with MRS1 phenotype were accompanied by increased immune cell infiltration. Analyses based on the GSE13507 dataset also yield consistent results (Supplementary Fig. 4C). In addition, immunomodulatory factors including chemokines, paired receptors, MHC molecules, and immunostimulator were also significantly up-regulated in the MRS1 phenotype in the TCGA-BLCA cohort (Supplementary Fig. 5A). Meanwhile, the gene markers of common immune cells such as CD8A and CD8B for CD8^+^ T cells, C1QA and MMP8 for macrophages, were also up-regulated in the MRS1 phenotype (Fig. 2F), consistent with previous research that chemokines and receptors recruit effector TIICs, including CD8^+^ T cells, macrophages, and antigen-presenting cells [51]. However, patients in the MRS1 subtype showed unfavorable prognosis, therefore we speculated that the MRS1 subtype suffered from the immuno-suppressive microenvironment. As expected, most immune checkpoints were highly expressed in patients with MRS1 phenotype, including CD274, PDCD1, and CTLA4 (Supplementary Fig. 5B). To verify the conclusions found in the TCGA-BLCA cohort, we explored the immune microenvironment characteristics of subtypes in the GSE13507 cohort. Immunomodulatory factors were also significantly up-regulated in the MRS1 phenotype (Supplementary Fig. 6A), and the expression of most conventional gene signatures of immune cells and immune checkpoints were remarkably increased in the MRS1 phenotype (Supplementary Fig. 6B). All these convince the results found in the TCGA-BLCA cohort. In summary, BLCA patients showed two heterogeneous metabolic subtypes with distinct features: MRS1, harboring inconspicuous metabolic characteristics but an immuno-suppressive TME; and MRS2, with upregulated lipid metabolism but a deserted TME, suggesting the diagnostic value in identifying the immunogenicity of BLCA.

### MRS1 phenotype patients belonged to the basal subtype and were sensitive to immunotherapy

**Fig. 3:**
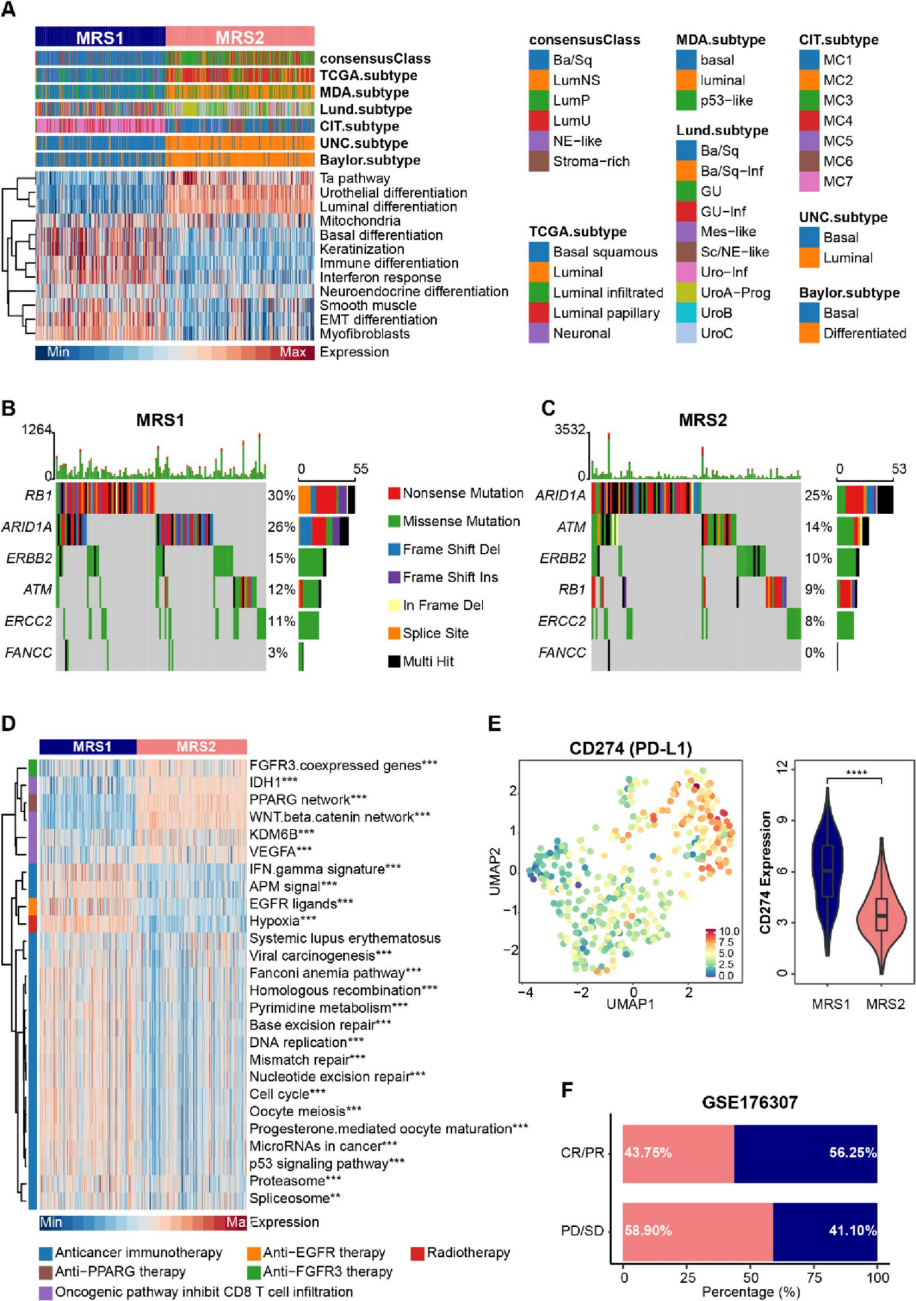
Metabolic phenotypes predicted molecular subtypes and clinical therapy. **A** Correlations between metabolic phenotypes and molecular subtypes using seven different algorithms (CIT, Lund, MDA, TCGA, Baylor, UNC, and consensus) and BLCA signatures. **B**, **C** Mutational profiles of chemotherapy-related genes in the MRS1 and MRS2 groups in the TCGA cohort. **D** Comparison of oncogenic pathways associated with therapeutic-targets between the MRS1 and MRS2 groups. *p < 0.05, **p < 0.01, ***p < 0.0001. **E** Left: UMAP visualization the expression distribution of PD-L1 (CD274). Right: Comparison the expression values of PD-L1 between the MRS1 and MRS2 groups. **F** Stacked histogram showing the percentage of MRS1 and MRS2 groups in CR/PR and PD/SD in GSE176307 cohort. CR: complete remission; PR: partial remission; PD: progression disease; SD: stable disease

To explore the response of MRS phenotype to clinical treatments, we assessed the molecular subtypes among patients in the TCGA-BLCA cohort, which has been proven as a prediction of clinical response [34, 52]. We found that patients with the MRS1 phenotype preferred to be the basal subtype consistently within seven established molecular subtyping systems, and possessed higher levels of basal differentiation (Fig. 3A), which was more likely to receive pathological response to immune checkpoint blockade (ICB) [32, 52]. Moreover, compared with patients harboring the MRS2 phenotype, patients with the MRS1 phenotype also had significantly higher mutation rates of RB1, ERBB2, and FANCC (Fig. 3B, C), which were associated with the response to neoadjuvant chemotherapy [53, 54], suggesting that the MRS1 patients might be more sensitive to neoadjuvant chemotherapy. Further analysis of therapy-predicted pathways based on the gene signatures showed that the enrichment scores of anti-cancer immunotherapy and radiotherapy-predicted pathways, as well as the EGFR ligands were remarkably higher in the MRS1 phenotype (Fig. 3D)

In line with results found in the TCGA-BLCA cohort, patients with the MRS1 phenotype in the GSE13507 dataset also tended to associate with basal-type BLCA (Supplementary Fig. 7A), and were enriched for anti-cancer immunotherapy pathways, radiotherapy-predicted pathways, and several immune checkpoints (Supplementary Fig. 6B, Supplementary Fig. 7B). According to the principle of immunotherapy, some immune checkpoints such as PD1/PD-L1 and CTLA4, can be targeted by inhibitors to enhance anti-cancer immunoreaction. Notably, compared with the MRS2 group, patients harboring the MRS1 phenotype had prominently higher expression values of immune checkpoints and immunotherapeutic targets (Supplementary Fig. 5B, Supplementary Fig. 6B), especially PD-L1 (Fig. 3E), a conventional drug-target of some well-known inhibitors, implying the potential immunotherapeutic sensitivity of the MRS1 group. Furthermore, we used an ICB treatment cohort dataset, the GSE176307 cohort, to further explore the association between metabolic phenotypes and immunotherapeutic response. The result showed that more than half of the patients receiving complete/partial remission after ICB had the MRS1 phenotype, while approximately 60% patients with ICB failure exhibited the MRS2 phenotype (Fig. 3F), suggesting that patients with the MRS1 phenotype may receive pathological remission after immunotherapy. Collectively, these results implied that ICB, neoadjuvant or adjuvant chemotherapy, and ERBB therapy can be considered, either alone or in combination, for the treatment of BLCA with the MRS1 phenotype.

### Tumor cells with higher proliferation and cholesterol biosynthesis contributed to the malignancy of the MRS1 phenotype

**Fig. 4:**
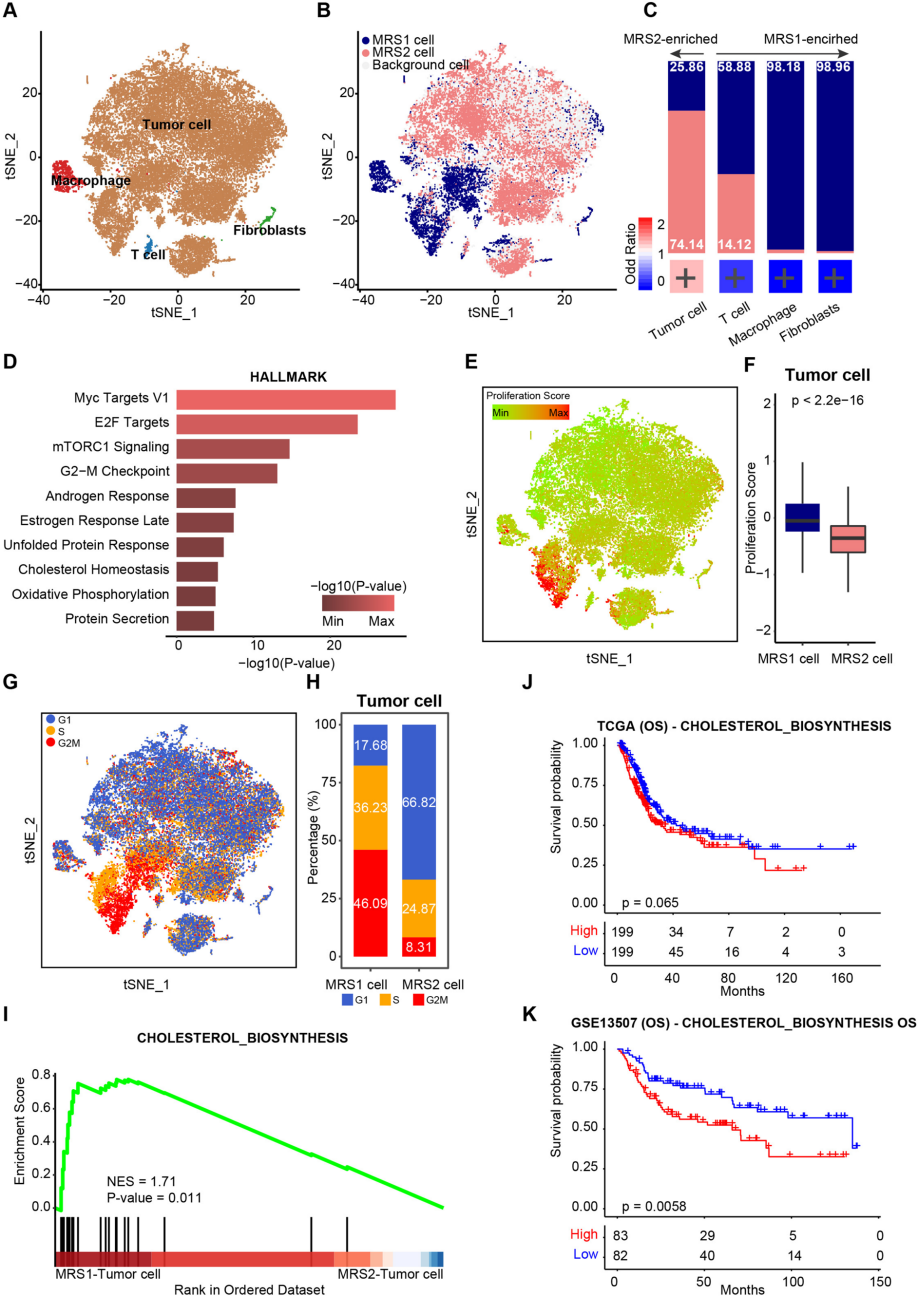
Comparison of the transcriptional patterns of MRS1 and MRS2 cells. **A** UMAP visualization of cell types annotated by classical gene markers. **B** UMAP visualization of cells with the MRS1 or MRS2 phenotype predicted by Scissor algorithm. Each dot represents one cells, colored by the groups. cells with the MRS1 phenotype genes are represented by navy, cells associated with the MRS2 phenotype are represented by lightcoral, and other cells are represented by lightgray. **C** Upper: Stacked histogram showing the percentage of MRS1 and MRS2 cells in each celltype. Bottom: Heatmap showing the enrichment of MRS1 cells in each cell type, with color encoded by odds ratio estimated by Fisher’s exact test. The red color represents enrichment of subpopulation in the MRS2 cells, while blue color represents depletion of subpopulation in the MRS cells. + p < 0.05. **D** Functional enrichment analysis of genes specifically expressed in the MRS1-tumor cells versus the MRS2-tumor cells. **E** A representative gene set enrichment analysis plot showing significant upregulated cholesterol biosynthesis in theMRS1-tumor cells versus the MRS2-tumor cells. **F** Proliferation scores overlaid on the UMAP embedding. **G** Comparison of proliferation scores between the MRS1- and MRS2- tumor cells. H UMAP visualization of G1 (blue), S (orange) and G2M (red) phases. I Fraction of G1 (blue), S (orange) and G2M (red) phases of the MRS1- and MRS2-tumor cells

In order to explore the transcriptional biomarkers of patients with MRS1 phenotype, we performed “Scissor”analysis to identify biologically and clinically relevant cell subpopulations from single-cell assays by leveraging phenotype and bulk-omics datasets. Firstly, we collected the scRNA-seq data of nine patients with BLCA from GEO datasets, including the GSE190888 [39] and the GSE186520 [40] datasets (Supplementary Fig. 8A). A total of 42,658 cells met the quality control criteria and were subsequently divided into 46 clusters by unsupervised clustering (Supplementary Fig. 8B). Based on the expression levels of well-established gene markers, we annotated the cell type for each cluster, including tumor cells, T cells, fibroblasts, and macrophages (Fig. 4A, Supplementary Fig. 8C). Then, we identified the MRS1 and MRS2 relevant cell subpopulations by leveraging the TCGA-BLCA dataset (Fig. 4B). Consistently, tumor cells were remarkably enriched in cells recognized as MRS2-related (MRS2 cells), while T cells, macrophages, and fibroblasts were significantly enriched in MRS1-related cells (MRS1 cells) (Fig. 4C). Functional enrichment analyses of HALLMARK database on the up-regulated genes of MRS1-tumor cells revealed a distinct functional pattern. As shown in Fig. 4D, the proliferation-related signaling pathways (G2-M checkpoint) and oxidative phosphorylation were significantly activated in MRS1-tumor cells, compared to MRS2-tumor cells. Consistently, we found that MRS1-tumor cells had significantly higher proliferation scores (Fig. 4E, F). Approximately 36% and 46% of the MRS1-tumor cells were in S and G2/M phase, respectively (Fig. 4G, H). Notably, cholesterol homeostasis was also significantly up-regulated in the MRS1-tumor cells versus the MRS2-tumor cells (Fig. 4D and I). We also found that patients with high cholesterol metabolism was associated with a poor OS in the TCGA and the GSE13507 cohorts (Fig. 4J, K), suggesting that the activation of cholesterol metabolism may lead to worse clinical outcomes. Summarily, our analysis implied that tumor cells with high proliferation and cholesterol biosynthesis contributed to the more malignant status of the MRS1 phenotype, perhaps in an immune-mediated mann

### S100A7 was a marker of MRS1-tumor cells

**Fig. 5:**
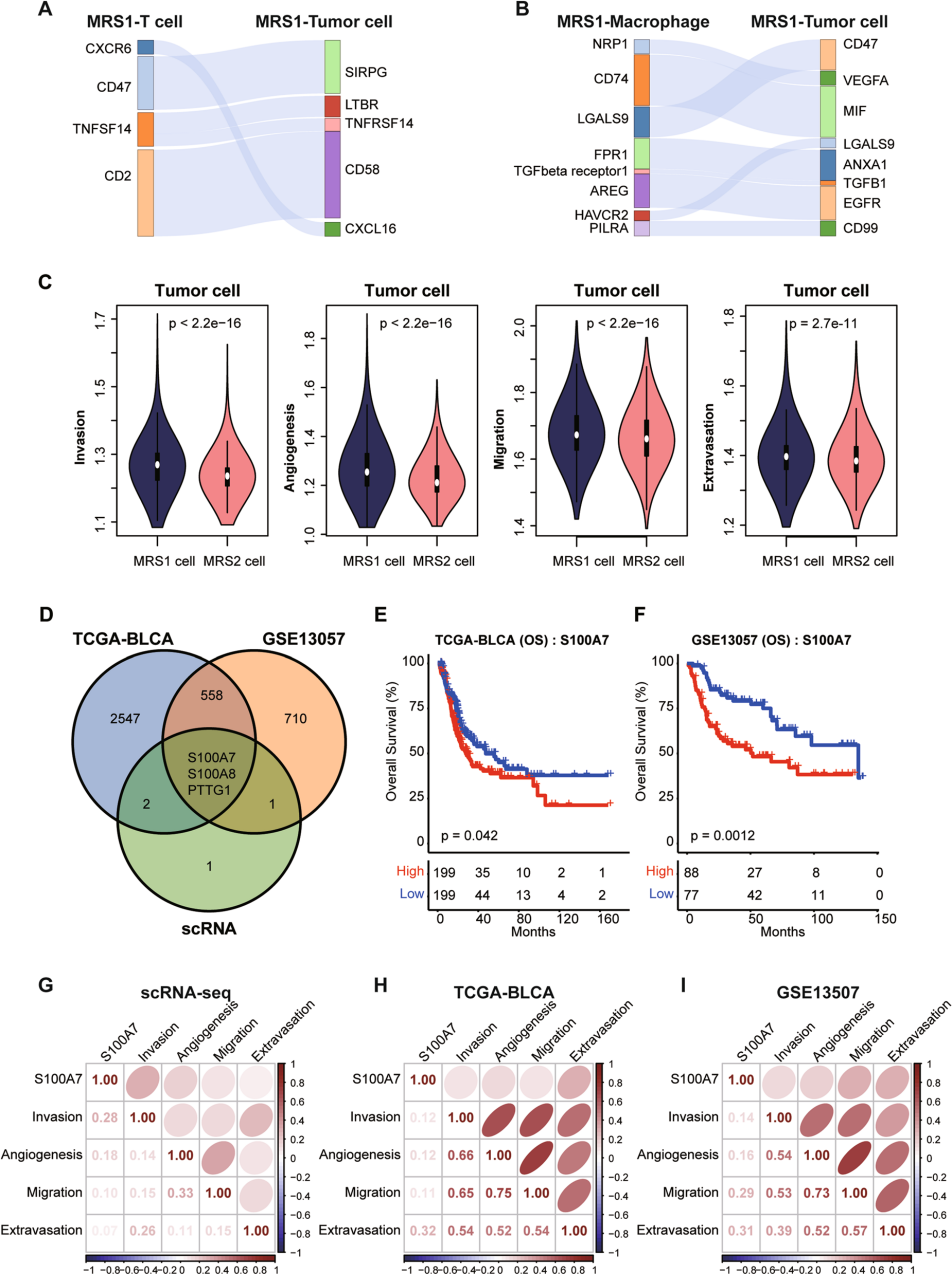
S100A7 was up-regulated in MRS1-tumor cells. **A** The interactions between MRS1-tumor cells and MRS1-T cells. **B** The interactions between MRS1-tumor cells and MRS1-macrophages. **C** Comparison of metastasis characteristics between the MRS1- and MRS2-tumor cells. **D** Venn plot showing the shared up-regulated genes in the MRS1-tumor cells and patients with the MRS1 phenotype. For TCGA-BLCA and GSE13057 cohort, the R package “limma” was applied. Genes with adjusted P-value< 0.05 and FC ≥ 1.5 were identified the DEGs of the MRS1 group. For single cell transcriptional dataset, “FindAllMarkers” function was used to identify the specific genes of MRS1- and MRS2-tumor cells. Genes with adjusted P-value< 0.05, FC ≥ 1.5, pct.1 ≥ 0.4 & pct.2 ≤ 0.1 were identified as the DEGs of the MRS1-tumor cells. **E**, **F** Kaplan-Meier analysis in term of OS of S100A7 in the TCGA-BLCA (**E**) and the GSE13057 (**F**) cohorts. All patients were categorized into two groups based on the median of the S100A7 expression. **G** Correlation between S100A7 and metastatic characteristics in the scRNA-seq dataset of MRS1 and MRS2-tumor cells. **H**, **I** Correlation between S100A7 and metastatic characteristics in the TCGA-BLCA (**H**) and GSE13507 cohorts (**I**)

The remarkably activated pathways in MRS1-tumor cells (e.g., cell-cycle signaling pathways and cholesterol metabolism) were essential in the tumor progression and the immunologic escape mediated by cell-cell communications [55–57]. Therefore, we hypothesize that the interactions between tumor cells and microenvironment cells would contribute to the malignant status of MRS1. Using “CellPhoneDB”, we performed a single-cell resolution cellular interactions analysis among MRS1 cell types identified in the above analyses (Supplementary Fig. 9A). Results showed that MRS1-tumor cells communicated with MRS1-T cells via CXCL16-CXCR6 (Fig. 5A), which has been reported to be involved in the T cell recruitment by tumor cells [58]. Besides, our results also found that some inhibitory interactions, such as SIRPG-CD47 and TNFRSF14-TNFSF14 interactions were detected between MRS1-tumor cells and MRS1-T cells (Fig. 5A). In addition, the interaction of LGALS9-HAVCR2, and ANXA1-FPR1 were also found between MRS1-tumor cells and MRS1-macrophages (Fig. 5B). Several research showed that the MIF-CD74 interaction was confirmed to involve in the persisting immunosuppressive M2 state of myeloid cells and abolished immune surveillance [59, 60]. Moreover, the MRS1-tumor cells also can communicate with the microenvironment cells via highly expressing VEGFA, a crucial regulator of pathological angiogenesis [61], and involve in the proliferation and metastasis of tumor cells [62]. Consistently, compared with the MRS2-tumor cells, the gene signatures of metastasis-related characteristics (invasion, angiogenesis, migration, and extravasation) were observably enriched in the MRS1-tumor cells (Fig. 5C). All results above supported that the tumor cells from patients with the MRS1 phenotype could enhance the malignancy of tumor via the cross-talk with microenvironment cells and the up-regulating of metastasis-related signaling pathways.

We then explored the crucial biomarkers of MRS1-tumor cells, finding that S100A7, S100A8, and PTTG1 were up-regulated in the MRS1 phenotype both in bulk data (TCGA-BLCA and GSE13057) and single-cell data (Fig. 5D, Supplementary Fig. 9B, Supplementary Tables 1, 3, 4). Notably, compared with PTTG1 and S100A8, approximately 50% MRS1-tumor cells expressed S100A7, with a 34-fold change than the expression percentage in MRS2-tumor cells (Figure S9C, D, Supplementary Table 4). Meanwhile, the expression levels of S100A7 were significantly associated with the worse outcomes both in the TCGA-BLCA and GSE13507 datasets (Fig. 5E, F, and Fig. S10). In addition, S100A7 were positively correlated with the enrichment of metastatic characteristics both at the single-cell and bulk omics levels (Fig. 5G and I), suggesting that S100A7 may correlate with the metastatic phenotype that observed in MRS1-tumor cells. Additionally, S100A7 also significantly positively correlated with the majority of immune features in these two cohorts (Supplementary Fig. 11A, B). Combined with the above results, we kindly proposed that the expression of S100A7 can characterize the inflamed TME, and predict the clinical outcomes of BLCA patients.

#### S100A7 was associated with poor prognosis and enhanced PD-L1 expression in the TMA cohort

**Fig. 6:**
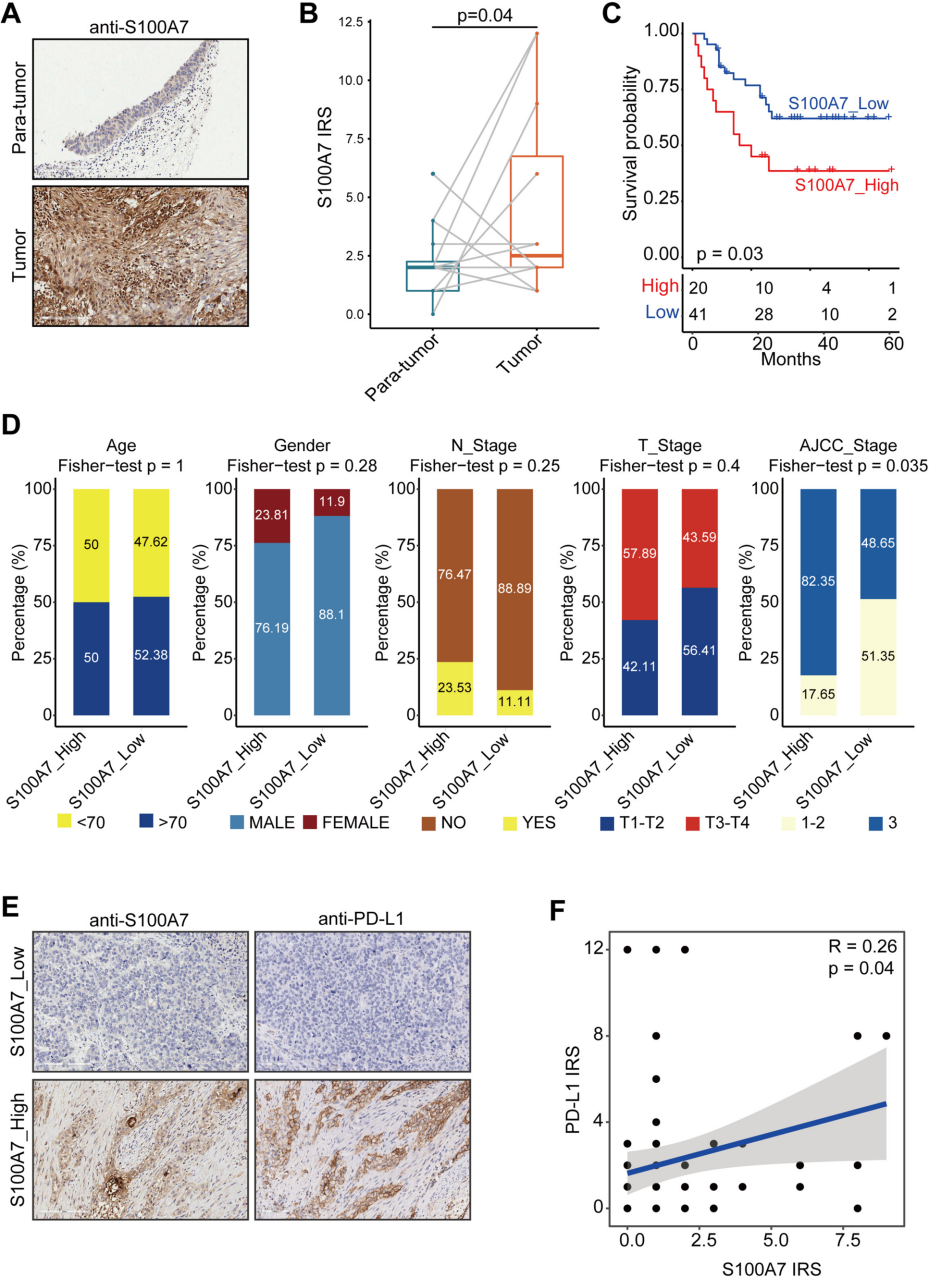
Validation of expression and prognostic value of S100A7 in the recruited TMA cohort. **A** Representative images revealing S100A7 expression in tumor and paratumor tissues using anti-S100A7 staining. Magnification, 200×. **B** Expression levels of S100A7 in tumor and paratumor tissues. **C** Kaplan-Meier analysis of S100A7 in term of OS in the TMA cohort. **D** Association between S100A7 expression and clinicopathological features in BLCA. **E** Representative images revealing PD-L1 expression in the high- and low-S100A7 groups. Magnification, 200×; (**F**) Correlation between S100A7 and PD-L1 expression

To confirm the above results, we also obtained a TMA cohort for validation, which included 63 BLCA and 16 para-cancerous samples. First of all, S100A7 was significantly upregulated in the BLCA tissues compared with para-cancerous tissues (Fig. 6A, B). Next, BLCA samples with S100A7 high expression showed remarkably poor prognosis (Fig. 6C). We used siRNA to knockdown S100A7 expression (Figure S12A), and the results showed that S100A7 knockdown significantly inhibited tumor cells proliferation (Figure S12B, C). In addition, we also explored the association between S100A7 expression and clinicopathological features. S100A7 was notably related to clinical stages, but not related to other features (Fig. 6D). Moreover, S100A7 was positively correlated with PD-L1 expression in the current cohort (Fig. 6E, F). Overall, based on the in-house cohort, we validated that S100A7 was correlated with poor prognosis and immuno-suppressive TME in BLCA.

## Discussion

In the present study, we have comprehensively explored the metabolic heterogeneity of patients with BLCA at transcriptional levels, and revealed that the metabolic phenotypes were associated with the immune infiltration microenvironment and clinical outcomes. Moreover, we elucidated that the metabolic phenotypes could accurately predict the molecular subtypes, and therapeutic response to several treatments, including ICB. Finally, we recognized a crucial biomarker: S100A7, which was positively correlated with many immune-related characteristics and associated with the poor clinical outcomes of BLCA patients. Importantly, the expression of S100A7 could predict the response to ICB.

With the complex genetic and transcriptional characteristics, BLCA is a kind of disease with highly inter-tumor heterogeneity [63], leading to about 17,100 deaths in the United States [64]. However, platinum-based chemotherapy is the standard-of-care-first-line used treatment strategy in a majority of BLCA [63]. Although approximately 60-70% patients were initially responding to platinum-based treatment, most of them will relapse and succumb to the disease due to the drug resistance [2]. Immunotherapy is an exciting breakthrough for the clinical treatment of patients. Several studies on immune checkpoint inhibitors (ICIs) have changed the treatment paradigm for BLCA patients [65]. However, the response rate of PD-1/PD-L1 inhibitors is only 20–24%, Most patients are insensitive to treatment and develop drug resistance [66]. Therefore, revealing the biological features for BLCA patients is important to development the precise therapy for individuals.

Increasing evidence has proved that the aberrant metabolic reprogramming of tumor cells is involved in the tumorigenesis and progression in many carcinomas [5, 6, 67]. Recent studies also underlined the inter- and intra-tumor heterogeneity and flexibility of metabolism in many solid tumors [14, 15], and revealed the crucial correlations between metabolic heterogeneity and microenvironment status, therapeutic response, and clinical outcomes [16, 18, 19]. Based on the limitation of precise therapy and the significance of metabolic reprogramming, we deconstructed the metabolic heterogeneity of BLCA and revealed the prognostic values of metabolic subtypes. Notably, some metabolic pathways, especially the lipid metabolism associated pathways, were significantly activated in patients with the MRS2 phenotype, while the MRS1 phenotype did not show distinct functional metabolic characteristics. Prognostic and immune microenvironment analysis showed that the MRS1 phenotype, with poor OS and PFS, preferred to shape an immuno-suppressive microenvironment, which might contribute to the enhanced malignancy of BLCA.

With the development of technology, scRNA-seq makes it possible to quantify the whole transcriptome at single cell level in a tissue mixture and provides an unprecedented opportunity to decipher the complexity of cellular heterogeneity and microenvironment [68]. A series of single-cell data analyses revealed that the tumor cells with the MRS1 phenotype had higher proliferation levels, activated cholesterol biosynthesis, and metastasis-related characteristics, which have been reported to contribute to the malignancy of BLCA [69]. Cholesterol metabolism shows the specificity of MRS1 subtype at the single cell level, but there is no difference in bulk data, which can be explained by the fact that the high mixed immune cells in bulk data weaken this feature of tumor cells. In addition, the cell-cell communications analysis showed that some interactions, involved in T cell recruitment, such as CXCL16-CXCR6; the formation of the immuno-suppressive microenvironment, such as LGALS9-HAVCR2, and ANXA1-FPR1; and promoting the proliferation and metastasis of tumor cells, were also detected between MRS1-tumor cells and MRS1-microenvironment cells. Collectively, these findings suggested the potential factors leading to the enhanced malignancy of BLCA.

Findings based on the PURE-01 study discovered that the basal-type BLCA showed the highest infiltration of immune cells, and better pathological response to pembrolizumab [52]. A consensus molecular classification of muscle-invasive bladder cancer also revealed a similar conclusion that basal-type tumors were more likely to receive pathological response to ICB [32]. Although the MRS1 phenotype had worse clinical outcomes, this subgroup preferred basal-type BLCA, a molecular subtype sensitive to immunotherapy and neoadjuvant chemotherapy. Metabolic subtyping for the immunotherapy cohort found that more than half of patients received complete remission/partial remission after ICB had the MRS1 phenotype, while approximately 60% patients failed to ICB exhibited the MRS2 phenotype, suggesting that patients with the MRS1 phenotype may be more sensitive to immunotherapy.

Prominently, we observed that S100A7 is mostly expressed on the MRS1-tumor cells, and can predict the clinical outcomes of BLCA patients. In addition, S100A7 was associated with the immuno-suppressive microenvironment. Lines of evidence have proved that S100A7 as a potential diagnostic and prognostic biomarker contributes to the malignancy of carcinomas via crosstalk and promoting angiogenesis [49].

## Conclusions

Taken together, our study reveals the metabolic heterogeneity and crucial biomarkers of BLCA based on the integrating analysis of bulk and scRNA-seq datasets. The group with the MRS1 phenotype had an immuno-suppressive TME and higher levels of S100A7, and preferred basal-type BLCA, which was sensitive to immunotherapy and neoadjuvant chemotherapy. Neoadjuvant chemotherapy, immunotherapy, or the combination treatment of the two are likely to benefit patients with the MRS1 phenotype. We anticipate that our study will provide important information for better understanding of the metabolic heterogeneity of BLCA, as well as provide a novel perspective for precision treatment.

### Supplementary Information

**Additional file 1: Supplementary Figure S1.** Related to Figure 1. BLCA samples had distinct metabolic gene expression from normal samples. (A) UMAP visualization of BLCA tumors and normal samples in the TCGA cohort for the expression of metabolic genes. (B) Global differences in metabolic gene expression between tumors and normal tissues in the TCGA-BLCA cohort. Left: The Euclidean expression distances were calculated between tumors and normal tissues (green), different samples of tumor tissues(red), and different samples of normal tissues (blue). The inset summarizes the average distances between pairs of tissues as a percentage of the average distance between tumors and normal tissues. ****p < 0.0001. Right: The correlation-based expression distances were calculated between tumors and normal tissues (green), different samples of tumor tissues (red), and different samples of normal tissues (blue). The inset summarizes the average distances between pairs of tissues as a percentage of the average distance between tumors and normal tissues. ****p < 0.0001. **Supplementary Figure S2.** Related to Figure 1. Consensus clustering matrixes of TCGA-BLCA patients using metabolic pathway enrichment score for k = 2 to k = 10. **Supplementary Figure S3.** Related to Figure 1. Comparison of the percentage of clinical characteristics between the MRS1 and MRS2 groups. **Supplementary Figure S4.** Related to Figure1 and Figure2. Metabolic-gene-based stratification of patients in the GSE13507 cohort. (A) UMAP visualization of metabolic subtypes in the TCGA cohort for the expression of metabolic genes. (B) Kaplan-Meier analysis in term of OS of TCGA BLCA patients. (C) Comparison of the ESTIMATE results between the MRS1 and MRS2 groups. **Supplementary Figure S5.** Related to Figure3. Comparison of Immune characteristics between the MRS1 and MRS2 groups in the TCGA-BLCA dataset. (A) Comparison of the immunomodulators (chemokines, immunostimulators, MHC, and receptors) enrichment scores between the MRS1 and MRS2 groups. (B) Expression levels of the immune checkpoints in the MRS1 and MRS2 groups. *p < 0.05, **p < 0.01, ***p < 0.0001. **Supplementary Figure S6.** Related to Figure3. Comparison of Immune characteristics between the MRS1 and MRS2 groups in the GSE13507 dataset. (A) Comparison of the immunomodulators (chemokines, immunostimulators, MHC, and receptors) enrichment scores between the MRS1 and MRS2 groups. (B) Expression levels of the gene signatures of TIICs and immune checkpoints in the MRS1 and MRS2 groups. *p < 0.05, **p < 0.01, ***p < 0.0001. **Supplementary Figure S7.** Related to Figure3. Metabolic phenotypes predicted molecular subtypes and clinical therapy in the GSE13507 cohort. (A) Correlations between metabolic phenotypes and molecular subtypes using seven different algorithms (CIT, Lund, MDA, TCGA, Baylor, UNC, and consensus) and BLCA signatures. (B) Expression levels of the gene signatures of TIICs and immune checkpoints in the MRS1 and MRS2 groups. *p < 0.05, **p < 0.01, ***p < 0.0001. **Supplementary Figure S8.** Related to Figure 4. Annotation of cell types. (A) UMAP visualization of 42,658 single cells from nine BLCA patients. (B) The unsupervised clustering of 42,658 cells. (C) Expression levels of known markers overlaid on the UMAP representation. **Supplementary Figure S9.** Related to Figure5. Ligand-receptor interactions between MRS1-tumor and MRS1-environment cells. (A) Heatmap showing the ligand-receptor interactions between MRS1-tumor and MRS1-environment cells. (B) Heatmap for gene expression levels of top 20 cell-type-specific genes. (C) UMAP visualization of S100A7 expressed genes. (D) Comparison of S100A7 expression between the MRS1- and MRS2-tumor cells. **Supplementary Figure S10.** Related to Figure 5. Kaplan-Meier analysis in term of OS in the TCGA-BLCA and the GSE13507 cohorts. All patients were categorized into two groups based on the median of the gene expression. **Supplementary Figure S11.** Related to Figure 5. Correlations between S100A7 expression and immune-related characteristics. (A) Left: Correlations between S100A7 expression and ESTIMATE results and immunomodulators (chemokines, immunostimulators, MHC, and receptors) enrichment scores in the TCGA cohort. Middle: Correlations between S100A7 expression and immune cell markers values in the TCGA cohort. Right: Correlations between S100A7 expression and immune checkpoints values in the TCGA cohort. (B) Left: Correlations between S100A7 expression and ESTIMATE results and immunomodulators (chemokines, immunostimulators, MHC, andreceptors) enrichment scores in the GSE13507 cohort. Middle: Correlations between S100A7 expression and immune cell markers values in the GSE13507 cohort. Right: Correlations between S100A7 expression and immune checkpoints values in the GSE13507 cohort. Significantly positive correlations are represented by orange, significantly negative correlations are represented by blue. **Supplementary Figure S12.** Related to Figure 6. S100A7 knockdown inhibits BLCA cells’ proliferation. (A) The transfected and silencing efficiency of S100A7 in BLCA cells was assessed by qRT-PCR. (B) The proliferative capacity of control and S100A7-silencing BLCA cells was examined by CCK-8 assay. (C) The proliferative capacity of control and S100A7-silencing BLCA cells was examined by EdU assay.

**Additional file 2: Supplementary Table S1.** The results of differentially expression analysis performed by R package "limma" in the TCGA-BLCA cohort. **Supplementary Table S2.** Related to Figure 2. Results of GSEA using gene sets of metabolic pathways comparing the MRS1 group *versus *MRS2 group in the TCGA cohort. **Supplementary Table S3.** The results of differentially expression analysis performed by R package "limma" in GSE13057. **Supplementary Table S4.** Highly expressed genes in MRS1- and MRS2-tumor cells.

## Data Availability

The GEO datasets used in this study (GSE13507, GSE176307, GSE190888, and GSE186520) can be downloaded from https://www.ncbi.nlm.nih.gov/gds. TCGA-BLCA gene matrix (HTSeq-FPKM) and clinical annotations can be obtained from https://xenabrowser.net.
