# Supplementary material for: A novel metabolic subtype with S100A7 high expression represents poor prognosis and immuno-suppressive tumor microenvironment in bladder cancer
Source: BMC Cancer. 2023 Aug 5;23:725. doi: 10.1186/s12885-023-11182-w (PMC10403905; doi:10.1186/s12885-023-11182-w)
Supplement: Supplementary file 1 — Additional file 1: Supplementary Figure S1. Related to Figure 1. BLCA samples had distinct metabolic gene expression from normal samples. (A) UMAP visualization of BLCA tumors and normal samples in the TCGA cohort for the expression of metabolic genes. (B) Global differences in metabolic gene expression between tumors and normal tissues in the TCGA-BLCA cohort. Left: The Euclidean expression distances were calculated between tumors and normal tissues (green), different samples of tumor tissues(red), and different samples of normal tissues (blue). The inset summarizes the average distances between pairs of tissues as a percentage of the average distance between tumors and normal tissues. ****p < 0.0001. Right: The correlation-based expression distances were calculated between tumors and normal tissues (green), different samples of tumor tissues (red), and different samples of normal tissues (blue). The inset summarizes the average distances between pairs of tissues as a percentage of the average distance between tumors and normal tissues. ****p < 0.0001. Supplementary Figure S2. Related to Figure 1. Consensus clustering matrixes of TCGA-BLCA patients using metabolic pathway enrichment score for k = 2 to k = 10. Supplementary Figure S3. Related to Figure 1. Comparison of the percentage of clinical characteristics between the MRS1 and MRS2 groups. Supplementary Figure S4. Related to Figure1 and Figure2. Metabolic-gene-based stratification of patients in the GSE13507 cohort. (A) UMAP visualization of metabolic subtypes in the TCGA cohort for the expression of metabolic genes. (B) Kaplan-Meier analysis in term of OS of TCGA BLCA patients. (C) Comparison of the ESTIMATE results between the MRS1 and MRS2 groups. Supplementary Figure S5. Related to Figure3. Comparison of Immune characteristics between the MRS1 and MRS2 groups in the TCGA-BLCA dataset. (A) Comparison of the immunomodulators (chemokines, immunostimulators, MHC, and receptors) enrichment scores between the MRS [file 12885_2023_11182_MOESM1_ESM.docx]

**Supplementary Figure S1.** Related to Figure 1. BLCA samples had distinct metabolic gene expression from normal samples. (A) UMAP visualization of BLCA tumors and normal samples in the TCGA cohort for the expression of metabolic genes. (B) Global differences in metabolic gene expression between tumors and normal tissues in the TCGA-BLCA cohort. Left: The Euclidean expression distances were calculated between tumors and normal tissues (green), different samples of tumor tissues (red), and different samples of normal tissues (blue). The inset summarizes the average distances between pairs of tissues as a percentage of the average distance between tumors and normal tissues. ****p < 0.0001. Right: The correlation-based expression distances were calculated between tumors and normal tissues (green), different samples of tumor tissues (red), and different samples of normal tissues (blue). The inset summarizes the average distances between pairs of tissues as a percentage of the average distance between tumors and normal tissues. ****p < 0.0001. **Supplementary Figure S2.** Related to Figure 1. Consensus clustering matrixes of TCGA-BLCA patients using metabolic pathway enrichment score for k = 2 to k = 10. **Supplementary Figure S3.** Related to Figure 1. Comparison of the percentage of clinical characteristics between the MRS1 and MRS2 groups. **Supplementary Figure S4.** Related to Figure1 and Figure2. Metabolic-gene-based stratification of patients in the GSE13507 cohort. (A) UMAP visualization of metabolic subtypes in the TCGA cohort for the expression of metabolic genes. (B) Kaplan-Meier analysis in term of OS of TCGA BLCA patients. (C) Comparison of the ESTIMATE results between the MRS1 and MRS2 groups. **Supplementary Figure S5.** Related to Figure3. Comparison of Immune characteristics between the MRS1 and MRS2 groups in the TCGA-BLCA dataset. (A) Comparison of the immunomodulators (chemokines, immunostimulators, MHC, and receptors) enrichment scores between the MRS1 and MRS2 groups. (B) Expression levels of the immune checkpoints in the MRS1 and MRS2 groups. *p < 0.05, **p < 0.01, ***p < 0.0001. **Supplementary Figure S6.** Related to Figure3. Comparison of Immune characteristics between the MRS1 and MRS2 groups in the GSE13507 dataset. (A) Comparison of the immunomodulators (chemokines, immunostimulators, MHC, and receptors) enrichment scores between the MRS1 and MRS2 groups. (B) Expression levels of the gene signatures of TIICs and immune checkpoints in the MRS1 and MRS2 groups. *p < 0.05, **p < 0.01, ***p < 0.0001. **Supplementary Figure S7. Related to Figure3.** Metabolic phenotypes predicted molecular subtypes and clinical therapy in the GSE13507 cohort. (A) Correlations between metabolic phenotypes and molecular subtypes using seven different algorithms (CIT, Lund, MDA, TCGA, Baylor, UNC, and consensus) and BLCA signatures. (B) Expression levels of the gene signatures of TIICs and immune checkpoints in the MRS1 and MRS2 groups. *p < 0.05, **p < 0.01, ***p < 0.0001. **Supplementary Figure S8. Related to Figure 4.** Annotation of cell types. (A) UMAP visualization of 42,658 single cells from nine BLCA patients. (B) The unsupervised clustering of 42,658 cells. (C) Expression levels of known markers overlaid on the UMAP representation. **Supplementary Figure S9**. Related to Figure5. Ligand-receptor interactions between MRS1-tumor and MRS1-environment cells. (A) Heatmap showing the ligand-receptor interactions between MRS1-tumor and MRS1-environment cells. (B) Heatmap for gene expression levels of top 20 cell-type-specific genes. (C) UMAP visualization of S100A7 expressed genes. (D) Comparison of S100A7 expression between the MRS1- and MRS2-tumor cells. **Supplementary Figure S10.** Related to Figure5. Kaplan-Meier analysis in term of OS in the TCGA-BLCA and the GSE13507 cohorts. All patients were categorized into two groups based on the median of the gene expression. **Supplementary Figure S11.** Related to Figure 5. Correlations between S100A7 expression and immune-related characteristics. (A) Left: Correlations between S100A7 expression and ESTIMATE results and immunomodulators (chemokines, immunostimulators, MHC, and receptors) enrichment scores in the TCGA cohort. Middle: Correlations between S100A7 expression and immune cell markers values in the TCGA cohort. Right: Correlations between S100A7 expression and immune checkpoints values in the TCGA cohort. (B) Left: Correlations between S100A7 expression and ESTIMATE results and immunomodulators (chemokines, immunostimulators, MHC, and receptors) enrichment scores in the GSE13507 cohort. Middle: Correlations between S100A7 expression and immune cell markers values in the GSE13507 cohort. Right: Correlations between S100A7 expression and immune checkpoints values in the GSE13507 cohort. Significantly positive correlations are represented by orange, significantly negative correlations are represented by blue. **Supplementary Figure S12.** Related to Figure 6. S100A7 knockdown inhibits BLCA cells’ proliferation. (A) The transfected and silencing efficiency of S100A7 in BLCA cells was assessed by qRT-PCR. (B) The proliferative capacity of control and S100A7-silencing BLCA cells was examined by CCK-8 assay. (C) The proliferative capacity of control and S100A7-silencing BLCA cells was examined by EdU assay.
